# Changes in Microbial Communities in Industrial Anaerobic Digestion of Dairy Manure Caused by *Caldicellulosiruptor* Pretreatment

**DOI:** 10.3390/biotech14030067

**Published:** 2025-08-28

**Authors:** Jakob Young, Maliea Nipko, Spencer Butterfield, Zachary Aanderud

**Affiliations:** 1 Department of Biology, Brigham Young University, Provo, UT 84602, USA; 2 Department of Microbiology and Molecular Biology, Brigham Young University, Provo, UT 84602, USA; mholden3@byu.edu; 3 Department of Sustainability & Renewable Energy Systems, University of Wisconsin-Platteville, Platteville, WI 53818, USA; spencerbutterfield8@gmail.com; 4 Department of Plant and Wildlife Sciences, Brigham Young University, Provo, UT 84602, USA; zachaanderud@gmail.com

**Keywords:** co-occurrence network, community connectivity, biological pretreatment, syntrophic methanogenesis, acetoclastic methanogenesis, hydrogenotrophic methanogenesis

## Abstract

Extremophilic biological process (EBP) pretreatment increases substrate availability in anaerobic digestion, but the effect on downstream microbial community composition in industrial systems is not characterized. Changes in microbial communities were determined at an industrial facility processing dairy manure in a modified split-stream system with three reactor types: (1) EBP tanks at 70–72 °C; (2) mesophilic Continuously Stirred Tank Reactors (CSTRs); (3) mesophilic Induced Bed Reactors (IBRs) receiving combined CSTR and EBP effluent. All reactors had a two-day hydraulic retention time. Samples were collected weekly for 60 days. pH, volatile fatty acid and bicarbonate concentrations, COD, and methane yield were measured to assess tank environmental conditions. Microbial community compositions were obtained via 16S rRNA gene sequencing. EBP pretreatment increased acetate availability but led to a decline in the relative abundance of acetoclastic *Methanosarcina* species in downstream IBRs. Rather, syntrophic methanogens, e.g., members of Methanobacteriaceae, increased in relative abundance and became central to microbial co-occurrence networks, particularly in association with hydrogen-producing bacteria. Network analysis also demonstrated that these syntrophic relationships were tightly coordinated in pretreated digestate but absent in the untreated CSTRs. By promoting syntrophic methanogenesis while increasing acetate concentrations, EBP pretreatment requires system configurations that enable acetoclast retention to prevent acetate underutilization and maximize methane yields.

## 1. Introduction

Anaerobic digestion (AD) of dairy manure relies on microbial metabolism to synthesize biogas consisting mainly of CO_2_(g) and CH_4_(g). The conversion of organic waste into biogas proceeds through four sequential microbial groups: (1) hydrolytic bacteria; (2) acidogenic bacteria; (3) acetogenic and syntrophic bacteria; (4) methanogenic archaea. The final step, methanogenesis, is performed by methanogenic archaea via three metabolic pathways. Primarily, acetoclastic methanogens, such as members of *Methanosarcina* and *Methanosaeta*, directly convert acetate into methane and bicarbonate in a single step catabolic reaction [1,2]:

CH_3_COO^−^(aq) + H_2_O(l) → CH_4_(g) + HCO_3_^−^(aq). (1)

Alternatively, hydrogenotrophic methanogenesis involves two distinct steps, reliant on a syntrophic partnership between hydrogenogenic bacteria and hydrogenotrophic methanogens [1]. The bacteria initially oxidize acetate to produce hydrogen:

CH_3_COO^−^(aq) + 4H_2_O(l) → H_2_CO_3_(aq) + HCO_3_^−^(aq) + 4H_2_(aq) (2)

Then, via direct interspecies hydrogen transfer, hydrogenotrophic methanogens consume the hydrogen and produce methane [1,3,4,5]:

H_2_CO_3_(aq) + 4H_2_(aq) → CH_4_(g) + 3H_2_O(l). (3)

As Reactions 2 and 3 sum to Reaction 1, and each produces bicarbonate, the net effect of methanogenic activity is an increase in pH. Hydrogenotrophic pathways can also involve conversion of volatile fatty acids (VFAs) other than acetate. For example, butyrate is oxidized by acetogenic bacteria according to [6]:

CH_3_CH_2_CH_2_COO^−^(aq) + H_2_O(l) + HCO_3_^−^(aq) → 2CH_3_COO^−^(aq) + CO_2_(aq) + 2H_2_(aq). (4)

CO_2_ and H_2_ are then converted into methane by hydrogenotrophic methanogens via Reaction 3. Accumulation of acetate and other longer-chain VFAs in digesters can inhibit Reaction 4 through product inhibition. To avoid inhibition, substantial activity of acetoclastic methanogens is necessary.

Finally, methylotrophic methanogens degrade methylated compounds, such as methanol, to produce methane [7,8]. Similarly to hydrogenotrophic methanogens, many methylotrophic methanogens require H_2_ for their activity, making them dependent on syntrophic interactions with bacteria [9]. Some methanogenic taxa, notably *Methanosarcina* spp., are metabolically flexible and can utilize a combination of acetoclastic, hydrogenotrophic, and methylotrophic methanogenesis pathways depending on metabolite concentrations and environmental conditions [10].

Digester operation is typically monitored by total VFA concentration, pH, and alkalinity, all of which affect microbial activity and metabolic pathways. A pH range of 6.8–7.5 is optimal for methanogenesis [11,12]. Note that VFAs produced by hydrolysis and acidogenesis all exist as anions at pH 6.8–7.5. Dairy manure is buffered near pH 7.8 by ammonium bicarbonate, so it is critical to also monitor bicarbonate concentration. However, alkalinity as measured by titration with HCl includes both bicarbonate and volatile fatty acid anions (VFAAs). Total VFA concentrations are commonly reported as acetate equivalents. For an accurate assessment of buffering capacity, the VFAA concentration must be subtracted from the measured alkalinity to estimate bicarbonate concentration.

Dairy manure contains large amounts of recalcitrant materials, including lignocellulose and peptidoglycans, whose incomplete hydrolysis may limit biogas yields from AD without pretreatment [13,14,15]. Although residual recalcitrant components do not diminish the value of digestate as fertilizer, their persistence reduces both energy recovery and potential financial return. In order to improve the cost-effectiveness of anaerobic digestion, a variety of pretreatment methods have been explored to break down recalcitrant materials before digestion [16]. Studies have shown that pretreatment methods reshape AD microbial communities by enhancing hydrolysis and altering the chemical characteristics of digestate [17]. Specifically, thermal hydrolysis pretreatment often reduces overall microbial diversity, enriches ammonia-tolerant hydrogenotrophic methanogens, and strengthens syntrophic interactions during the anaerobic digestion of protein-rich substrates [18,19]. Consequently, hydrogenotrophic methanogenesis typically becomes the dominant form of methane generation under extreme pretreatment conditions, although pretreated reactors continue to produce methane through both acetoclastic and hydrogenotrophic pathways [17]. Metagenomic analyses of thermally pretreated sludge reactors demonstrated that higher temperatures correspond with a decrease in alpha diversity, while microbial networks become more interconnected [18].

The novel use of the extremophilic biological process (EBP), which combines hyperthermophilic temperatures with hydrolytic activity from *Caldicellulosiruptor* species, has been shown to greatly increase the rates of hydrolysis and acidogenesis, leading to greater biogas yields [6,20,21]. Pretreatment with *Caldicellulosiruptor bescii* has been reported in previous studies to double biogas yield, which would likely deliver returns several times greater than the added cost of EBP implementation in an industrial AD facility [20]. *Caldicellulosiruptor* species use exozymes to catalyze hydrolysis of lignin, celluloses, and peptidoglycans [22,23,24]. The saccharides released from celluloses are primarily catabolized into acetate. For example:

Cellulose + H_2_O(l) → C_6_H_12_O_6_(aq), (5)

C_6_H_12_O_6_(aq) + 3HCO_3_^−^(aq) → 3CH_3_COO^−^(aq) + 3H_2_CO_3_(aq), (6)

H_2_CO_3_(aq) → H_2_O(l) + CO_2_(g). (7)

EBP has shown promise in laboratory studies but remains uncommon in full-scale operations, partly due to limited quantitative data on yield improvement, effects on microbial activity, and overall operational performance. Most research therefore has been confined to bench scale or single step pretreatment studies, leaving the longitudinal evolution of microbial communities and network connectivity in full scale multi-reactor digestion systems largely unexplored. As a result, plant engineers lack the microbial insights needed to optimize retention times, reactor configurations, and pretreatment strategies at industrial scale.

To address these limitations, we monitored microbial and chemical parameters at a commercial digestion facility with an existing modified split-stream EBP pretreatment system. We had no involvement in the system’s design and collected data only during standard plant operation. At the facility, dairy manure was treated in EBP tanks inoculated with *Caldicellulosiruptor* species and maintained at elevated temperatures (70–72 °C) to promote hydrolysis and acidogenesis. In parallel, mesophilic Continuously Stirred Tank Reactors (CSTRs) were fed raw manure without pretreatment. Effluents from both streams were combined and subsequently processed in mesophilic Induced Bed Reactors (IBRs), combining acetate-rich digestate from pretreatment with an active acetoclastic community of methanogens from untreated digestate in the CSTRs. IBRs are fixed-film anaerobic digesters that retain biomass on support media, enabling high solids and microbial retention while maintaining shorter liquid hydraulic retention time (HRT) [25]. The HRT in all tanks was two days, as a means of maximizing throughput and maintaining continuous substrate availability. However, the low HRT likely impacted microbial retention by washout, especially of slow-growing methanogens. We expect the microbial relative abundance (RA) to represent a steady-state system determined by HRT, not abundances determined by growth limitations [26,27].

Given the combination of elevated temperatures and enhanced enzymatic hydrolysis provided by EBP tanks inoculated with *Caldicellulosiruptor* species, we hypothesized four primary outcomes: (1) EBP pretreatment would increase lignocellulose hydrolysis rates, elevating acetate concentration in the digestate going into the IBRs. (2) The extreme thermal conditions in EBP tanks would force a microbial community shift, promoting thermotolerant methanogens, such as *Methanothermobacter* spp. and high temperature-tolerant bacterial populations. (3) Microbes that form biofilms would be favored due to the retention of particulate matter by the design of IBRs. (4) The abundance of free-living acetoclastic methanogens would be depleted due to washout.

## 2. Materials and Methods

### 2.1. Site Description

**Figure 1:**
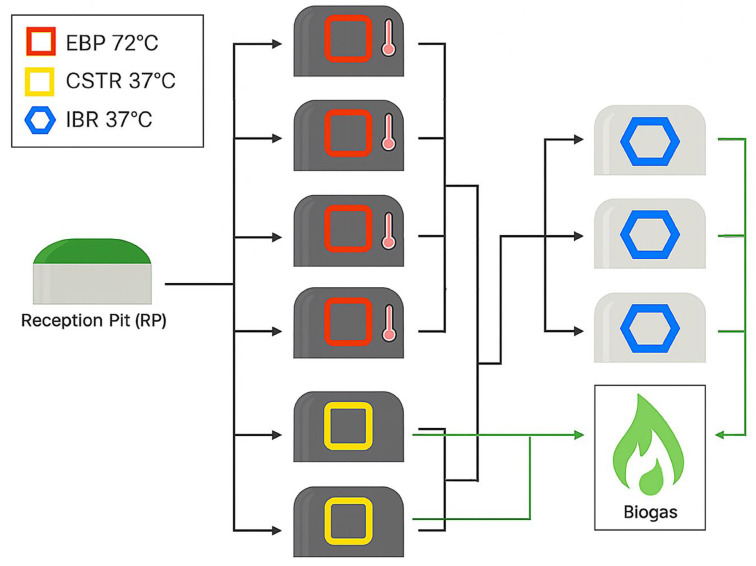
Schematic of the studied facility. Raw manure from the reception pit is fed into parallel CSTRs and EBP tanks, with effluents from both streams combined and directed into the IBRs. Biogas was collected and analyzed from both the CSTRs and the IBRs.

The study was conducted at a commercial facility with nine 189 cubic meter anaerobic reactors, see Figure 1. Dairy manure was collected in a reception pit (RP) and fed into four EBP tanks at 70–72 °C and two CSTRs at 35–37 °C. The effluent from all six initial tanks was mixed and fed into three IBRs at 35–37 °C. Each reactor had real-time monitoring of temperature, total biogas, and biogas methane content on site. Hydraulic retention time in the EBP tanks, CSTRs, and IBRs was approximately two days. The dairy manure feedstock averaged 83.95 g/L total solids and 65.91 g/L volatile solids (*n* = 11), while the plant’s biogas contained an average of 54.4 ± 1.7% methane (*n* = 15) over the monitoring period. Total methane production was calculated as the product of total biogas and percent methane. The EBP tanks were inoculated with a mixture of *C. bescii*, *C. owensensis*, *C. acetigenus*, and *C. saccharolyticus*.

### 2.2. Cultivation of Caldicellulosiruptor spp. and Inoculum Preparation

All four *Caldicellulosiruptor* spp. were acquired as lyophilized pellets from the German Collection of Microorganisms and Cell Cultures GmbH (DSMZ, Braunschweig, Germany). Each culture was revived anaerobically following DSMZ guidelines until the colony reached approximately 2.5 × 10^6^ cells/mL. Specifically, *C. bescii* (DSMZ #6725) was grown in Medium 516 (DSMZ_Medium516.pdf), while *C. owensensis* (DSMZ #13100), *C. saccharolyticus* (DSMZ #8903), and *C. acetigenus* (DSMZ #12137) were each cultivated on Medium 640 (DSMZ_Medium640.pdf). Freezer stocks of each culture were prepared by mixing 500 µL of cell suspension (≈2.5 × 10^6^ cells/mL) with 500 µL of 60% glycerol in a Don Whitley Scientific miniMACS anaerobic chamber (West Yorkshire, UK). The resulting mixtures were gently agitated at room temperature for 10 min, allowing the glycerol to permeate the cells, and then stored at –80 °C. For inoculum production, 2 mL each of *C. owensensis*, *C. acetigenus*, *C. saccharolyticus*, and *C. bescii* were added to 1 L of Medium 640 under anaerobic conditions in a miniMACS workstation, followed by incubation at 75 °C until the population reached ≈ 2.5 × 10^6^ cells/mL. Cell density for all cultures was determined by microscopy. Each EBP tank received 2 L of inoculum for initial seeding.

### 2.3. Sample Collection and Storage

40 milliliters of sample were gathered from the effluent of each tank approximately weekly over 60 days. To ensure representative sampling, the reactor sample port was flushed prior to collection. Samples were frozen and transported in insulated packaging to the lab at BYU in Provo, Utah for chemical and biological analyses. Samples were kept frozen at −20 °C until analysis. Note that this processing of samples likely increased the measured pH by removing CO_2_ from the solution.

### 2.4. Analyses of Samples

A Hannah HI98195 probe (Hannah Instruments, Woonsocket, RI, USA) was used to measure pH. VFAs and chemical oxygen demand (COD) were measured with Hach TNT 872 Vial Tests and Hach TNT 822 Vial Tests, respectively (Hach, Loveland, CO, USA). The Hach TNT Vial Test for VFA measures total VFA and reports the result as acetate equivalents. For both VFA and COD measurements, each sample was subsampled, centrifuged for 10 min at 4000 RPM, and filtered with an Avantor VWR Syringe Filter (Glass fiber, 13 mm, 1.0 μm) (Avantor, Radnor Township, PA, USA). Because VFA concentrations in AD effluent tend to be too large for measurement by the Hach TNT Vial Test, each subsample was diluted 10-fold with Milli-Q ultrafiltered water prior to analysis.

Alkalinity was quantified via titration to pH 4 using 1 M HCl on a TitraLab AT1000 (Hach Instruments; Loveland, CO, USA). Samples were diluted 100-fold with Milli-Q ultrapure water before titration. Because the measured alkalinity includes both bicarbonate and VFAAs, alkalinity was converted to an estimate of bicarbonate concentration (mg/L) with the equation:

(8) $$\frac{mgHCO_{3}^{-}}{L}=\;\left[\frac{Alkalinity}{50\;mg\;per\;equivalent}+\frac{Total\;VFA}{59\;mg\;per\;equivalent}\right]\;\;\times \;61\;mg\;per\;equivalent.$$

Alkalinity is expressed as mg CaCO_3_ equivalents per liter and total VFA is expressed as mg acetate per liter. The constants 50 and 59 represent the mg of CaCO_3_ and acetate that neutralize one mmole of H^+^ ions. The factor 61 is the molar mass of bicarbonate and is used to convert mmol to mg.

### 2.5. DNA Extraction and Sequencing

DNA was isolated from 300 mg of each frozen sample using the Qiagen DNEasy PowerWater Kit Pro (Qiagen, Kilden, Germany). Purity and concentration of the extracted DNA were checked with a Nanodrop One Microvolume UV-Vis Spectrophotometer (Thermo Fisher Scientific, Waltham, MA, USA). The 16S rRNA regions coded by the DNA were amplified for sequencing on an Illumina MiSeq platform (Illumina, San Diego, CA, USA), conducted at the Utah State University Sequencing Center in Logan, UT (https://cores.utah.edu/dna-sequencing/ (accessed on 8 July 2025)). The V4 hypervariable region of the 16S rRNA gene was targeted using primers 515FB and 806RB, producing ~420 bp amplicons suitable for taxonomic profiling of bacterial and archaeal communities. First-round PCR reactions were performed in triplicate using Platinum II Hot Start master mix, followed by pooling and 1:50 dilution for indexing (ThermoFisher Scientific, Waltham, MA, USA). A second PCR added unique dual indices (p5/p7) to each sample (Integrated DNA Technologies, Coralville, IA, USA), and products were purified using SeraPure magnetic beads (Cytiva, Marlborough, MA, USA). Amplicon size was verified (~420 bp) using a TapeStation or Fragment Analyzer (Agilent Technologies, Santa Clara, CA, USA), and samples were pooled stoichiometrically and sequenced on an Illumina platform (Illumina, San Diego, CA, USA).

### 2.6. DNA Sequence Processing

All 16S sequences were analyzed using the Qiime2 2022.2 pipeline [28]. First, raw reads were demultiplexed and subjected to quality filtering, removing chimeric reads and applying default denoising parameters via the DADA2 pipeline [29]. To standardize across samples, data were rarefied to 80,000 reads per sample. We built phylogenetic trees of denoised ASVs using fasttree2 [30]. ASVs were grouped at 97% similarity and assigned taxonomy via local BLAST (v2.16.0) against the SILVA taxonomic database [28,31]. Note that some species will not be detected or identified by the SILVA database [32]. Additionally, note that DNA presence in a tank does not denote activity [33]. All analyses were conducted at the ASV level and then aggregated to the genus or phylum level for visualization and interpretation.

For community dissimilarity, Bray–Curtis distances were calculated and visualized with Principal Coordinate Analysis (PCoA). Using the Qiime2R functionality within the tidyverse package, PCoA plots were generated in R v4.2.0 [34]. Permutational multivariate analysis of variance (PERMANOVA) using the adonis2 function in the vegan package further assessed the statistical significance of between-group differences [35].

### 2.7. Microbial Correlation Testing

Spearman correlations of all taxa abundance and environmental variables (COD, VFA, bicarbonate, pH, total methane production, and tank temperature) were determined. Spearman correlation was used because the microbial abundance data was non-normally distributed, often skewed, and compositional. Specifically, for each microbial taxon, abundance of the taxon was tested against COD, VFA, bicarbonate, pH, total methane production, and tank temperature across all samples from all tanks throughout the entire 60-day experiment. This allowed a comprehensive approach to correlation analysis, calculating correlation across the entirety of our data, rather than constraining the correlation to tanks that had changing environmental variables over time. In order to minimize Type I errors given the large number of correlations being tested, the *p*-values for these tests were adjusted using the Benjamini–Hochberg procedure.

### 2.8. Microbial Co-Occurrence Networks

The microbial co-occurrence networks were calculated using the cooccur package in R v4.2.0 [36]. All detected co-occurrences were filtered for significance (*p* < 0.05). Taxa unidentified by the SILVA database were removed from the analysis. Networks were calculated using the Fruchterman–Reingold method and a minimum distance was set between nodes to prevent overlap [37]. Distance between nodes was set to the inverse of effect size and the size of nodes was set to node connectivity. The calculated network was plotted using ggraph on R [38]. Connectivity, eigenvector values, and betweenness were calculated for each node. Modularity and connectivity statistics were computed for each graph.

## 3. Results and Discussion

### 3.1. Community Compositions

#### 3.1.1. Reception Pit (RP) Community

**Figure 2:**
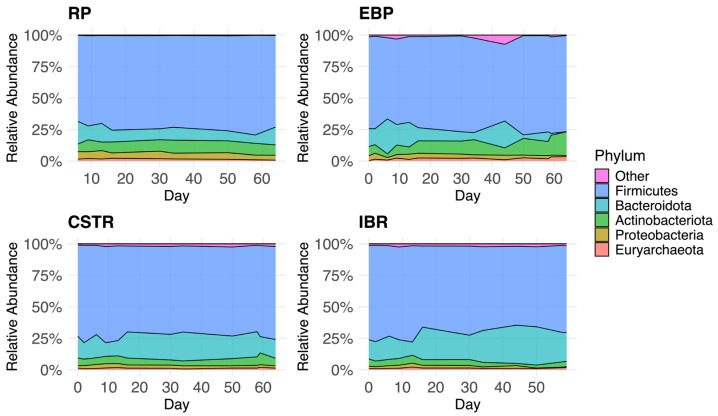
Stacked area plots showing relative abundance of the five most common microbial phyla in each tank type over time. The abundance values represent the average across all tanks within each category by date. See Table 1. Bacteroidota relative abundance decreases in the EBP tanks and increases in the IBRs. Actinobacteria relative abundance increases in the EBP tanks.

**Figure 3:**
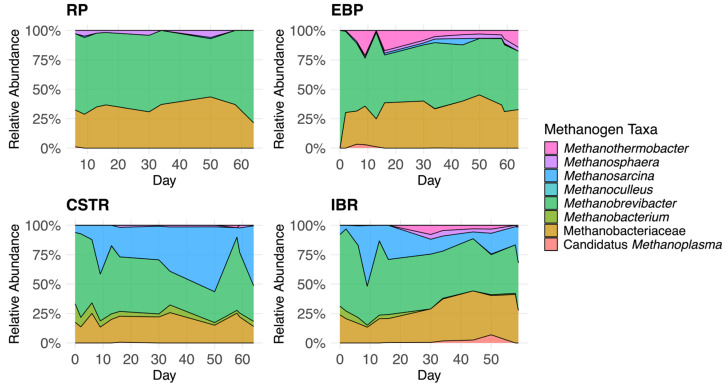
Stacked area plot depicting the community abundance of methanogenic archaea over time across the three tank categories and the Reception Pit (RP). Abundances are averages across all tanks within the category on that date. See Table 2. Hydrogenotrophic methanogens dominate all communities. Acetoclastic *Methanosarcina* relative abundance grows in the CSTRs and declines in the IBRs. Methylotrophic and thermophilic methanogen relative abundance grows in the IBRs.

The RP tank accepts raw dairy manure that has a stable microbial community (Figure 2 and Figure 3, Table 1 and Table 2). Hydrogenotrophic *Methanobrevibacter* (63.7 ± 2.3%) and members of Methanobacteriaceae (33.5 ± 2.0%) together dominated the methanogen community. *Methanosarcina* genus (0.3 ± 0.2%), the only genus detected in the manure capable of acetoclastic methanogenesis, was extremely low in RA. Prior comparative metagenomic analysis observed that hydrogenotrophic archaea typically dominate the archaeal population within raw manure, while acetoclastic populations remain minor [39]. *Methanosphaera* comprised an average RA of 2.4% of the methanogenic community in the raw manure (Table 2).

#### 3.1.2. Continuously Stirred Tank Reactor (CSTR) Community

The CSTRs, fed manure from the RP, exhibited stable microbial communities of Firmicutes (72.0 ± 0.8%), Bacteroidota (20.8 ± 1.2%), and Actinobacteriota (4.3 ± 0.3%). The main methanogenic taxa were *Methanobrevibacter* (47.8 ± 3.5%), *Methanosarcina* (25.6 ± 4.0%), and members of Methanobacteriaceae (19.9 ± 1.2%). The abundance of *Methanosarcina*, which were essentially absent in the RP, increased substantially upon entering the CSTRs relative to the hydrogenotrophic methanogens that were overwhelmingly dominant in the RP (Wilcoxon rank-sum, *p* < 0.001). Within the CSTRs, *Methanosarcina* RA continued to rise over time (Spearman ρ = 0.49, *p* < 0.05). *Methanosarcina* was correlated with lower VFA (Spearman’s ρ = −0.35, *p* < 0.01, n = 103) and higher bicarbonate (Spearman’s ρ = 0.55, *p* < 0.001, n = 103) concentrations. In the CSTRs, therefore, *Methanosarcina* species actively facilitated acetoclastic methanogenesis, consuming VFAs and releasing bicarbonate, consistent with Reaction 1.

*Methanosarcina* played a central role in the methanogenic community within the CSTRs, with significant proliferation despite the system’s short HRT. *Methanosarcina* are known to dominate acetoclastic communities in low-HRT reactors, likely because some species can form multicellular aggregates that reduce washout in well-mixed reactors [40]. Additionally, members of *Methanosarcina* exhibit faster acetate consumption kinetics and higher tolerance for elevated acetate concentrations, allowing them to outcompete hydrogenotrophic methanogens under acetate-rich conditions [40,41]. These traits, along with their metabolic flexibility to use acetoclastic, hydrogenotrophic, and methylotrophic pathways of methanogenesis, likely explain their proliferation from trivial levels in the RP to a large proportion of the methanogenic community in the CSTRs.

*Methanosarcina* species were the only archaeal taxa capable of acetoclastic methanogenesis detected in the system, making them solely responsible for the downstream conversion of the increased acetate from EBP into methane. This has important design implications for facilities using EBP and highlights the need to optimize upstream mesophilic reactor conditions to encourage *Methanosarcina* growth before seeding downstream reactors. If *Methanosarcina* populations fail to establish or decline, the acetate generated by EBP pretreatment would remain unconverted, undermining the intended benefits of the process.

#### 3.1.3. Extremophilic Biological Process (EBP) Tank Community

*Firmicutes* abundance was constant, comprising the majority of microbes in these tanks (72.9 ± 1.1%), Figure 2. The presence of the Thermotogota and Fusobacteriota phyla, which fall under the other category in Figure 2, were noted on occasion. Fusobacteriota specializes in degradation of plant biomass such as cellulose and cellobiose and likely contributed to the hydrolysis and metabolism of recalcitrant compounds in the EBP tanks [42]. Thermotogota are known to prefer higher temperatures and produce H_2_, possibly forming relationships with syntrophic methanogens [43,44]. In this case *Methanosarcina* was undetected in the EBP tanks, likely because the growth of these archaea are inhibited at high temperatures [45,46]. As noted above, the split-stream configuration delivers CSTR effluent into the IBRs precisely to replenish the mesophilic acetoclastic methanogens inactivated by the hyperthermophilic EBP pretreatment.

As seen in Table 1, the hyperthermophilic EBP reactors have a larger average RA of Euryarchaeota (2.2 ± 0.2% of total) compared with the CSTRs and IBRs. This is explained by two sources of methanogen DNA. Microorganisms from the RP have died or gone dormant and their genetic material is detected [47]. Additionally, thermophilic methanogens, e.g., *Methanothermobacter* spp. (6.2 ± 1.5% of methanogens in EBP), from the raw manure grew in the EBP tanks. Notably, the RA of *Methanothermobacter* was higher than in the RP (Wilcoxon rank-sum, *p* < 0.01) and increased with time (Spearman ρ = 0.37, *p* < 0.05). The growth in RA of *Methanothermobacter* genus and detection of members of Thermotogota phylum confirms our hypothesis that hyperthermophilic pretreatment selects for thermotolerant methanogens and bacteria. Although the relative abundance of thermophilic taxa increased during the study period, their persistence in extended operation will depend on maintaining hyperthermophilic conditions, adequate substrate availability, and tolerance to ammonia. Note that *Caldicellulosiruptor* species were present but not included in the analysis.

#### 3.1.4. Induced Bed Reactor (IBR) Community

In the IBR tanks, the relative abundance of Bacteroidota increased over time (Spearman ρ = 0.73, *p* < 0.001) relative to *Firmicutes* (ρ = –0.73, *p* < 0.001) and Actinobacteriota (ρ = –0.55, *p* < 0.001), both of which declined. The dominant methanogenic taxa in the IBRs were *Methanobrevibacter* (48.7 ± 2.4%), *Methanosarcina* (16.8 ± 2.5%), and members of Methanobacteriaceae (26.5 ± 1.7%). The RA of *Methanosarcina* genus in the IBRs was smaller than that of the CSTRs (Wilcoxon rank-sum, one-sided *p* = 0.050). Nevertheless, the CSTR effluent supplied to the IBRs successfully introduced an active acetoclastic community of *Methanosarcina* species, albeit at lower abundance relative to other syntrophic methanogenic taxa that became more prevalent in the IBRs. Namely, the syntrophic methanogens that had higher RA in the IBRs than in the CSTRs included members of Methanobacteriaceae (Wilcoxon rank-sum, *p* < 0.01), *Methanothermobacter* (Wilcoxon rank-sum, *p* < 0.01), and Candidatus *Methanoplasma* (Wilcoxon rank-sum, *p* < 0.05). The RA of both Candidatus *Methanoplasma* (Spearman’s ρ = 0.48, *p* < 0.001, n = 54) and *Methanothermobacter* (Spearman’s ρ = 0.48, *p* < 0.001, n = 54) correlated with higher methane production in the IBRs. These findings suggest a growth of alternative methanogenesis pathways relative to the classic acetoclastic pathway in digestate following EBP. The evidence from the IBRs indicates EBP pretreatment shifts the methanogenic community away from acetoclastic methanogenesis, likely because IBRs retain large solid particles but allow soluble substrates, such as acetate, to pass through underutilized due to low HRT.

### 3.2. Co-Occurrence Networks

#### 3.2.1. Reception Pit (RP) Network

**Figure 4:**
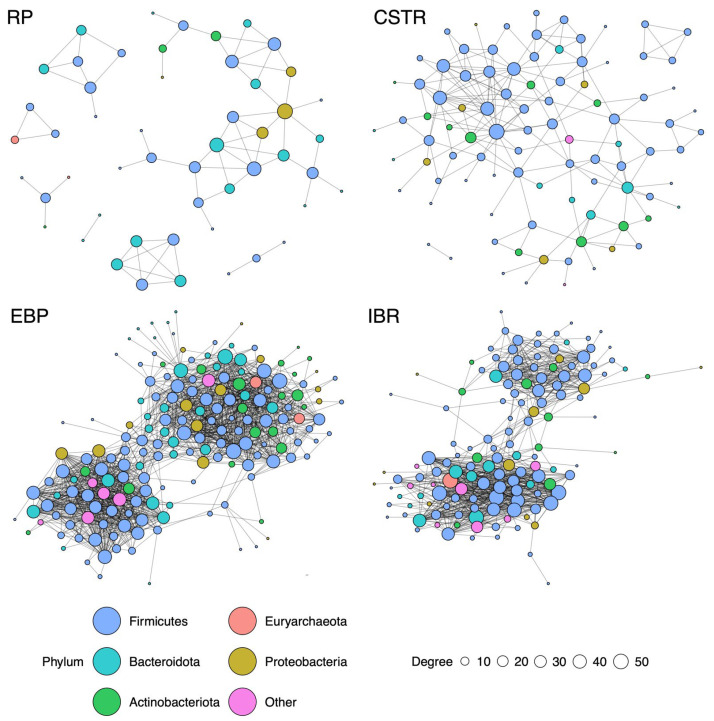
Co-occurrence network graphs depicting community relationships between microbial taxa in the RP (*n* = 10), EBP tanks (*n* = 37), IBRs (*n* = 33), and CSTRs (*n* = 23). Lines are drawn between taxa when they are observed together in samples significantly more frequently than random (*p* < 0.01). The closeness between a pair of taxa corresponds with the effect size of the relationship. The size of the node represents the number of connections to other taxa. See Table 3 for network statistics. EBP and IBR networks are dense and interconnected, with hydrogenotrophic methanogens central to their communities. Raw manure (RP) and CSTR communities are disconnected and indistinct. No co-occurrence relationships with methanogenic taxa were detected in the CSTR tanks.

The RP co-occurrence network was distinct due to its high modularity of 0.74 (Table 3). The community is loosely organized and contains many disconnected groups of taxa (Figure 4). Methylotrophic *Methanosphaera* and hydrogenotrophic *Methanobrevibacter* are noted in the manure community network but are relatively disconnected and possess low eigenvector values. The average taxon had a positive relationship with 2.7 other taxa.

#### 3.2.2. Continuously Stirred Tank Reactor (CSTR) Network

The CSTR network had a modularity value of 0.57 and consisted of 3 components (Table 3). The most influential taxa with the highest eigenvector values were entirely of the *Firmicutes* phylum. Despite having an average RA of 1.3% (Table 1) in the CSTR tanks, no positive relationships toward methanogenic taxa were detected. Many known acetogenic taxa, such as members of Lachnospiraceae and *Syntrophomonas*, presented with high betweenness factors [48,49]. Acetogenic taxa therefore played a key role in this community, transforming the products of related hydrolytic and acidogenic bacteria into acetate. The lack of hydrogenotrophic methanogens in the network suggests a fundamental limitation to methane production due to their reliance on interspecies hydrogen transfer [4].

#### 3.2.3. Extremophilic Biological Process (EBP) Tank Network

The EBP pretreatment tank co-occurrence network exhibited a highly dense and interconnected community (Figure 4). Each taxon in the EBP tanks was connected to an average of 16.6 other taxa (Table 3). The network had a modularity value of 0.5. Members of Firmicutes and Bacteroidota, including *Herbinix* and *Petrimonas* genera, comprised the majority of taxa with high eigenvector values. Notably, the third most influential taxon in the EBP tanks was a member of the Verrucomicrobiota phylum, presenting with an eigenvector of 0.995. The role of Verrucomicrobiota is largely uncharacterized in the context of AD. Methylotrophic *Methanosphaera* and hydrogenotrophic *Methanobrevibacter* displayed centrality in the community, both exhibiting high eigenvector and degree values. Both *Methanosphaera* and *Methanobrevibacter* exhibited strong co-occurrence relationships with several syntrophic bacteria capable of H_2_ production, notably members of Thermoanaerobacteraceae, Thermovenabulum, and *Caldicoprobacter* [50,51,52].

In thermophilic reactors, enhanced breakdown of proteins can lead to increased free ammonia accumulation [53,54]. When ammonia levels rise under high-temperature conditions, acetoclastic pathways can be suppressed and digestion relies more heavily on syntrophic interspecies hydrogen transfer [55,56]. It is therefore unsurprising that, *Methanosarcina*, the only genus capable of acetoclastic methanogenesis detected in the system, was detected in very low abundance (Table 2) and did not display positive co-occurrence with any identified bacterial taxa. In addition to acetoclastic suppression, both thermophilic temperatures and short retention times in AD have been seen to cause a shift towards hydrogenotrophic methanogenesis [5,57]. In this case, increased free ammonia from hyperthermophilic conditions under EBP and the brief HRT hindered acetoclastic pathways and shifted the community towards syntrophic methanogenic networks.

#### 3.2.4. Induced Bed Reactor (IBR) Network

In the IBRs processing the mixed CSTR and EBP effluents, the co-occurrence network exhibited a modularity of 0.43 and a mean connectivity of 11.69 taxa per node (Table 3). *Methanosphaera* displayed both the highest betweenness and a high eigenvector score (0.8), marking it as a keystone species that sustained a highly interconnected syntrophic web with diverse bacterial partners. In particular, due to its hydrogen dependency, *Methanosphaera* formed strong relationships with syntrophic bacteria known to produce H_2_, such as members of Thermoanaerobacteraceae, *Defluviitoga*, and *Caldicoprobacter* [50,51,58,59].

The persistence of these syntrophic taxa in the IBR network, originally structured under hyperthermophilic EBP pretreatment, highlights the resilience of the hydrogen-dependent community when conditions shifted to mesophilic temperatures. No methanogens capable of acetoclastic methanogenesis presented in the co-occurrence network. Despite elevated VFAA concentrations in the IBRs, no difference was discerned between the bicarbonate concentrations of the CSTRs and IBRs (Tukey, Δ = −198.53, *p* > 0.05). Under active acetoclastic conversion of the surplus acetate (Reaction 1), we would expect to see elevated bicarbonate levels in the IBRs. Acetoclastic pathways were therefore insufficient to convert the excess acetate into additional methane and bicarbonate, suggesting that elevated methane yields in these mesophilic reactors relied on the enduring syntrophic interactions formed during thermophilic pretreatment. The continued centrality of syntrophic taxa in the IBR networks, including *Methanosphaera*, supports our hypothesis that biofilm-forming microorganisms are favored under IBR retention conditions.

#### 3.2.5. Comparison of Networks

The RP network was the most disconnected, exhibiting the highest modularity (0.74) and lowest mean connectivity value (2.7). Despite slightly higher connectivity, the CSTR network remained structurally similar to the RP. In contrast, the EBP and IBR communities exhibited much higher connectivity and resembled each other. The EBP tanks have the highest connectivity value (16.6) of all tanks. Both the EBP and IBR networks consisted of 1 component, while the RP and CSTR consisted of multiple components. The EBP and IBR networks have clustering coefficients of 0.6, larger than both the RP (0.44) and CSTRs (0.38). While the most influential members of the CSTR network were entirely Firmicutes, the most influential taxa in both the EBP and IBR networks were more diverse, consisting of Firmicutes, Bacteroidota, and others.

The syntrophic webs assembled during hyperthermophilic pretreatment persisted into the IBRs, demonstrating the resilience of hydrogenotrophic partnerships even under mesophilic operation. The persistence of these tightly connected syntrophic webs into downstream reactors suggests potential for stable long-term operation. However, the detection of the genetic material of these taxa does not confirm metabolic activity, so persistence in the IBRs does not necessarily mean the entire network remains alive or functional [33]. Over longer time scales, community drift or loss of key syntrophic partners could disrupt hydrogen transfer efficiency. Monitoring network connectivity metrics during extended operation may provide early warning of destabilization.

Except the mesophilic CSTRs processing raw manure, syntrophic methanogens exhibited relationships with other taxa in all other networks. Acetoclastic *Methanosarcina* species did not have co-occurrence relationships in any of the tanks due to the high levels of acetate from the RP and EBP pretreatment. Together, these patterns indicate that EBP pretreatment causes large changes in downstream microbial networks, favoring tightly connected syntrophic communities and diminishing the role of free-living acetoclastic methanogen species. From an operational standpoint, connectivity and community structure may be useful indicators of functional dominance, guiding adjustments to operational parameters in order to maximize methane production.

### 3.3. Community Dissimilarity and Relationship with Environmental Variables

**Figure 5:**
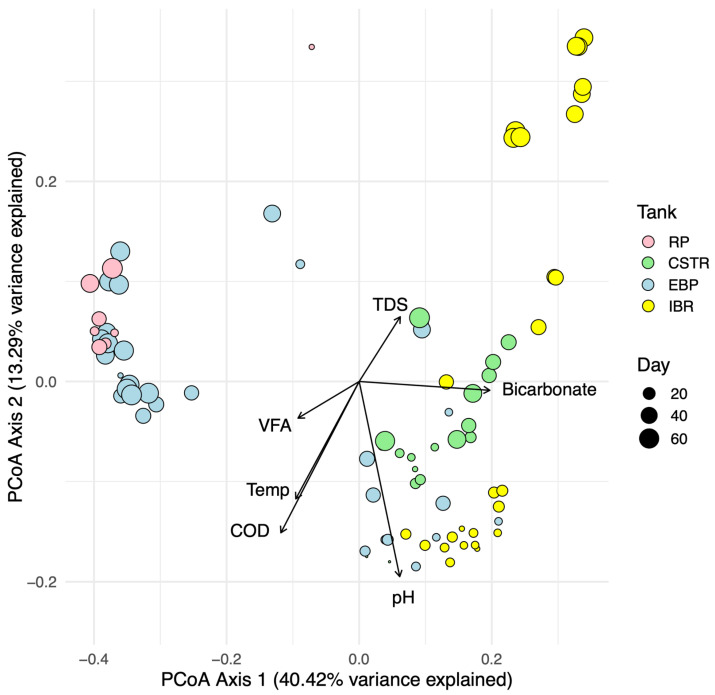
Principal Coordinates Analysis (PCoA) biplot of microbial community composition in all samples with colors corresponding to tank type. Point sizes indicate when the sample was collected with smaller points represent samples collected near the beginning of the experiment while the larger points represent samples collected near the end of the experiment. A point represents the microbial community composition of a sample, i.e., all of the detected taxa in a sample weighted according to their relative abundance. Vectors depict significant correlations with environmental variables and microbial community compositions. The length of each vector indicates the strength of the correlation with each of the environmental variables, total dissolved solids (TDS), volatile fatty acids (VFA), temperature (Temp), pH, and bicarbonate. Reactors responsible for methane production (CSTRs and IBRs) and tanks responsible for pretreatment and storing manure (EBP tanks and RP) are separated by Axis 1.

Dissimilarities between microbial community compositions, i.e., all of the detected taxa in a sample weighted according to their RA, were assessed between tank types using PCoA (Figure 5). The strength and direction of the correlations among significant environmental variables and community compositions are overlaid as vectors. Microbial communities differed by tank type (PERMANOVA, R^2^ = 0.36, *p* < 0.001) due to large differences in environmental conditions between tank types. Axis 1, with 40.42% variance explained, largely differentiated communities by VFA and bicarbonate concentrations. Additionally, axis 1 horizontally separated the reactors responsible for methane production (CSTRs and IBRs) from the raw manure and pretreatment tanks (RP and EBP tanks). Axis 2, with 13.29% variance explained, vertically differentiated communities by pH, COD, temperature, and TDS.

**Figure 6:**
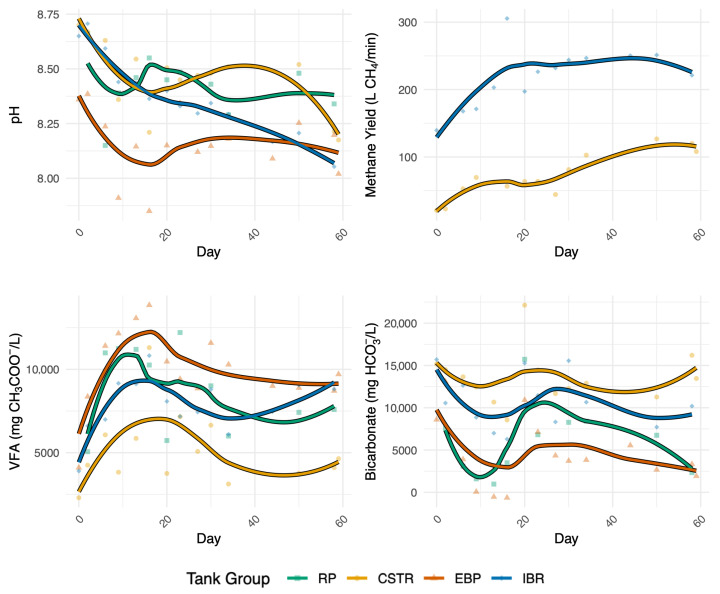
Trends in pH, total VFA, bicarbonate, and methane gas flow (L/min) by tank type over time. Regressions are calculated and drawn using the Locally Estimated Scatterplot Smoothing (LOESS) technique to clearly illustrate trends in variables over time. EBP tanks consistently had lower pH and bicarbonate, and higher total VFAA concentrations than other tanks. IBRs exhibited decreasing pH and increasing VFAA concentrations over time. CSTRs had higher bicarbonate concentrations and lower total VFA concentrations than other tanks. IBRs consistently produced more methane on average than the CSTRs.

The trajectory of the EBP tanks over time aligns with expected *Caldicellulosiruptor* metabolic activity. Near the beginning of the experiment, communities in the EBP tanks were associated with higher COD, temperature, and pH. But these communities progressively became associated with high VFA and low bicarbonate concentrations as time passed, consistent with *Caldicellulosiruptor* metabolism outlined in Reactions 5, 6, and 7. This trend is supported in the environmental data, with the EBP tanks showing significantly lower pH (Welch *t*-test, one-sided, *p* < 0.001) and higher acetate concentrations than raw manure in the RP, in line with the expected accumulation of VFAAs from *Caldicellulosiruptor* activity (Figure 6). In fact, VFAA concentrations in the EBP tanks frequently exceeded 10,000 mg/L and were significantly greater than those in the CSTR (Δ = 4797.614, *p* < 0.001) and IBR tanks (Δ = 1793.985, *p* < 0.05).

Although a simultaneous decrease in pH and increase in acetate were expected outcomes of EBP pretreatment, sustained low pH can inhibit methanogenic activity, particularly for acetoclastic methanogens that are more sensitive than hydrogenotrophic taxa [5,12]. However, the facility’s split-stream design likely mitigated potential inhibition by blending EBP and CSTR effluents before entering the IBRs. Blending of effluents may serve as a long-term strategy to buffer downstream pH in EBP systems, supporting methane synthesis despite elevated acetate levels.

Movement of the CSTR and IBR tanks on the dissimilarity matrix demonstrates methanogenic activity. Specifically, CSTR and IBR communities were notably different from those of the RP and EBP and were distinctly correlated with higher bicarbonate concentrations, indicating active methanogenic communities, i.e., see Reactions 1, 2, and 3. Although pH in the CSTR tanks was comparable to that of the raw manure, across both CSTR and IBR reactors pH was negatively correlated with methane production (Pearson r = −0.45, *p* < 0.001; Spearman rho = −0.49, *p* < 0.001), further supporting the link between elevated VFA availability and increased functional performance. Additionally, bicarbonate concentrations varied significantly among tank types (ANOVA, F(3,25) = 10.10, *p* < 0.001), with EBP tanks exhibiting substantially less bicarbonate than both the CSTRs (Δ = −3584.87, *p* < 0.001) and IBRs (Δ = −3386.34, *p* < 0.001).

At the start of the experiment, the CSTRs and IBRs were similar (Figure 5), but the IBR communities became distinct due to introduction of EBP effluent, dominated by thermophiles not suitable for activity in the mesophilic IBRs. Consequently, the microbial community that developed in the IBRs over time was shaped by both the physiochemical characteristics of the EBP effluent and the community in the CSTR effluent, ultimately resulting in a community distinct from both the EBP and CSTR communities. Because the mesophilic IBRs in this system received thermally treated effluent, the microbial and chemical composition of their feed differed from conventional mesophilic digestion, which may influence optimal operating conditions and warrants further optimization for heat-treated substrates. The divergence of IBR communities over time highlights how upstream pretreatment and reactor design directly shaped downstream function and community structure. As the experiment progressed, IBR pH declined steadily (−0.0089 units per day, R^2^ = 0.834, *p* < 1 × 10^−10^), and pH strongly influenced community composition (envfit r^2^ = 0.714, *p* = 0.001). Compared to CSTRs, the IBRs had significantly higher VFAA concentrations (Wilcoxon rank-sum, *p* < 0.001) and significantly lower bicarbonate concentrations (Wilcoxon rank-sum, *p* < 0.001) throughout the experiment (Figure 6). These findings are explained by a large increase in syntrophic activity, which replaces bicarbonate with acetate as seen in Reaction 2. While the IBR tanks exhibited higher methane yields than the CSTRs (Wilcoxon rank-sum, *p* < 0.001), the large concentration of acetate received from EBP pretreatment remained critically underutilized (Figure 6). The brief HRT in the IBR tanks prevented the growth and accumulation of acetoclastic methanogens responsible for the conversion of acetate into bicarbonate which would stabilize pH (Reaction 1). If acetoclastic methanogens had been able to effectively convert the abundant acetate in the IBRs, methane yields would likely have been even higher, consistent with previous reports of up to a 100% increase when *Caldicellulosiruptor* pretreatment is fully effective [17].

## 4. Conclusions

EBP pretreatment caused acetate accumulation and elevated methane production in downstream IBRs, as originally hypothesized. The continual seeding of the acetoclastic community, namely *Methanosarcina* species, from the mesophilic CSTRs was successful in introducing these taxa within the IBRs and contributed to the observed elevated methane yields. Despite this continuous input, however, the abundance of the *Methanosarcina* genus declined relative to the abundance of syntrophic methanogens in the IBRs. Network analysis revealed that EBP and IBR communities were more cohesive and functionally integrated than those in CSTRs, suggesting more robust syntrophic methanogenesis pathways within pretreated digestate. Overall, higher methane yields in IBRs appeared to result from both the presence of *Methanosarcina* from the CSTRs and the enhanced activity of syntrophic methanogens promoted by EBP.

The results indicate that EBP increases acetate availability while shifting downstream communities toward syntrophic pathways that largely bypass acetate. To counter this imbalance and fully utilize the available acetate, system designers should pair pretreatment with reactor configurations and retention times that support the growth and retention of slow-growing methanogenic taxa, especially acetoclasts. Continual seeding of downstream reactors with a strong acetoclastic community, coupled with extended HRT, is essential to ensure acetate utilization and sustained acetoclastic activity in EBP-treated digestate. Additionally, targeted alkalinity adjustment following EBP, such as by bicarbonate addition or effluent blending, may support conditions that optimize acetoclastic activity and prevent an inhibitory decline in pH. As these findings are specific to the studied facility’s configuration and conditions, future work should experimentally investigate whether such strategies support long-term community stability and function in other systems with EBP implementation.

## Figures and Tables

**Table 1:** Summary of non-methanogenic community composition by mean relative abundance of phyla across samples, separated by RP (n = 10), EBP tanks IBRs (n = 33), and CSTRs (n = 23). Since the relative abundance is averaged from all samples throughout the experiment, standard error is included. See Figure 2.

Phylum	RP 1 Tank, *n* = 10	EBP 4 Tanks, *n* = 37	CSTR 2 Tanks, *n* = 23	IBR 3 Tanks, *n* = 33
Firmicutes	66.8 ± 6.5%	72.9 ± 1.1%	72.0 ± 0.8%	70.3 ± 0.9%
Bacteroidota	11.6 ± 1.2%	10.4 ± 1.6%	16.9 ± 1.0%	20.8 ± 1.2%
Actinobacteriota	9.7 ± 1.2%	10.5 ± 0.9%	5.8 ± 0.3%	4.3 ± 0.3%
Proteobacteria	9.6 ± 4.6%	2.6 ± 0.2%	2.5 ± 0.1%	1.8 ± 0.2%
Euryarchaeota	1.4 ± 0.2%	2.2 ± 0.2%	1.3 ± 0.1%	1.3 ± 0.1%
Other	0.8 ± 0.4%	1.5 ± 0.4%	1.5 ± 0.1%	1.5 ± 0.1%

**Table 2:** Summary of methanogen community composition by mean relative abundance across samples, separated by tank type. The relative abundance is presented as the percentage of the methanogenic community. Since the relative abundance is averaged from all samples throughout the experiment, standard error is included. See Figure 3.

Methanogen	RP 1 Tank, *n* = 10	EBP 4 Tanks, *n* = 37	CSTR 2 Tanks, *n* = 23	IBR 3 Tanks, *n* = 33
*Methanobrevibacter* spp.	63.7 ± 2.3%	56.3 ± 2.7%	47.8 ± 3.5%	48.7 ± 2.35%
Members of *Methanobacteriaceae*	33.5 ± 2.0%	33.5 ± 1.7%	19.9 ± 1.2%	26.5 ± 1.74%
*Methanosarcina* spp.	0.3 ± 0.2%	1.2 ± 0.4%	25.6 ± 4.0%	16.8 ± 2.49%
*Methanothermobacter* spp.	ND ^1^	6.2 ± 1.5%	0.1 ± 0.1%	1.6 ± 0.57%
*Methanobacterium* spp.	ND	ND	5.5 ± 0.9%	2.4 ± 0.5%
*Methanosphaera* spp.	2.4 ± 0.7%	2.0 ± 0.3%	0.5 ± 0.2%	1.4 ± 0.4%
Members of Candidatus *Methanoplasma*	0.1 ± 0.1%	0.4 ± 0.2%	ND	1.0 ± 0.4%

^1^ ND = Not Detected.

**Table 3:** Co-occurrence network summary statistics by tank type. Components refer to the number of disconnected groups of taxa in a community. Modularity measures how well a network divides into communities with dense internal and sparse external connections. Mean Connectivity is the average number of taxa each taxon is connected to within a network. Mean clustering represents the degree to which nodes in a network tend to cluster together into tight groups. Means are presented ± SE.

Parameter	RP	EBP	CSTR	IBR
Components	7	1	3	1
Modularity	0.74	0.51	0.57	0.44
Mean Connectivity	2.70 ± 0.21	16.58 ± 1.00	3.93 ± 0.32	11.70 ± 0.94
Mean Clustering	0.44 ± 0.05	0.60 ± 0.02	0.38 ± 0.04	0.60 ± 0.03

## Data Availability

Raw 16S rRNA gene sequencing data and associated metadata are publicly available through the NCBI BioProject database under accession number PRJNA1311259.

## Abbreviations

The following abbreviations are used in this manuscript:

RP	Reception Pit
CSTR	Continuously Stirred Tank Reactor
EBP	Extremophilic Biological Process
IBR	Induced Bed Reactor
AD	Anaerobic Digestion
VFA	Volatile Fatty Acid
VFAA	Volatile Fatty Acid Anion
COD	Chemical Oxygen Demand
TDS	Total Dissolved Solids
RA	Relative Abundance
HRT	Hydraulic Retention Time

## References

[1] Kurade M.B., Saha S., Salama E.-S., Patil S.M., Govindwar S.P., Jeon B.-H. (2019). Acetoclastic methanogenesis led by *Methanosarcina* in anaerobic co-digestion of fats, oil and grease for enhanced production of methane. Bioresour. Technol..

[2] Jiang Y., Banks C., Zhang Y., Heaven S., Longhurst P. (2018). Quantifying the percentage of methane formation via acetoclastic and syntrophic acetate oxidation pathways in anaerobic digesters. Waste Manag..

[3] Stams A.J.M., Plugge C.M. (2009). Electron transfer in syntrophic communities of anaerobic bacteria and archaea. Nat. Rev. Microbiol..

[4] Junicke H., Feldman H., Van Loosdrecht M.C.M., Kleerebezem R. (2016). Limitation of syntrophic coculture growth by the acetogen. Biotechnol. Bioeng..

[5] Demirel B., Scherer P. (2008). The roles of acetotrophic and hydrogenotrophic methanogens during anaerobic conversion of biomass to methane: A review. Rev. Environ. Sci. Biotechnol..

[6] Hansen L.D. (2023). Thermodynamic method for analyzing and optimizing pretreatment/anaerobic digestion systems. Biofuel Res. J..

[7] Bueno de Mesquita C.P., Wu D., Tringe S.G. (2023). Methyl-Based Methanogenesis: An Ecological and Genomic Review. Microbiol. Mol. Biol. Rev..

[8] Glass J.B., Whitman W.B., Gargaud M., Irvine W.M., Amils R., Cleaves H.J., Pinti D., Cernicharo Quintanilla J., Viso M. (2019). Methanogenesis. Encyclopedia of Astrobiology.

[9] Harirchi S., Wainaina S., Sar T., Nojoumi S.A., Parchami M., Parchami M., Varjani S., Khanal S.K., Wong J., Awasthi M.K. (2022). Microbiological insights into anaerobic digestion for biogas, hydrogen or volatile fatty acids (VFAs): A review. Bioengineered.

[10] De Vrieze J., Hennebel T., Boon N., Verstraete W. (2012). *Methanosarcina*: The rediscovered methanogen for heavy duty biomethanation. Bioresour. Technol..

[11] Atasoy M., Cetecioglu Z. (2022). The effects of pH on the production of volatile fatty acids and microbial dynamics in long-term reactor operation. J. Environ. Manag..

[12] Franke-Whittle I.H., Walter A., Ebner C., Insam H. (2014). Investigation into the effect of high concentrations of volatile fatty acids in anaerobic digestion on methanogenic communities. Waste Manag..

[13] Atelge M.R., Atabani A.E., Banu J.R., Krisa D., Kaya M., Eskicioglu C., Kumar G., Lee C., Yildiz Y.Ş., Unalan S. (2020). A critical review of pretreatment technologies to enhance anaerobic digestion and energy recovery. Fuel.

[14] Bandgar P.S., Jain S., Panwar N.L. (2022). A comprehensive review on optimization of anaerobic digestion technologies for lignocellulosic biomass available in India. Biomass Bioenergy.

[15] Nguyen V.K., Chaudhary D.K., Dahal R.H., Trinh N.H., Kim J., Chang S.W., Hong Y., Duc La D., Nguyen X.C., Ngo H.H. (2021). Review on pretreatment techniques to improve anaerobic digestion of sewage sludge. Fuel.

[16] Amin F.R., Khalid H., Zhang H., Rahman S.U., Zhang R., Liu G., Chen C. (2017). Pretreatment methods of lignocellulosic biomass for anaerobic digestion. AMB Express.

[17] Pasalari H., Gharibi H., Darvishali S., Farzadkia M. (2024). The effects of different pretreatment technologies on microbial community in anaerobic digestion process: A systematic review. J. Environ. Health Sci. Eng..

[18] Zhang L., Gong X., Wang L., Guo K., Cao S., Zhou Y. (2021). Metagenomic insights into the effect of thermal hydrolysis pre-treatment on microbial community of an anaerobic digestion system. Sci. Total Environ..

[19] Shi J., Zhang G., Zhang H., Qiao F., Fan J., Bai D., Xu G. (2022). Effect of Thermal Hydrolysis Pretreatment on Anaerobic Digestion of Protein-Rich Biowaste: Process Performance and Microbial Community Structures Shift. Front. Environ. Sci..

[20] Hansen J.C., Aanderud Z.T., Reid L.E., Bateman C., Hansen C.L., Rogers L.S., Hansen L.D. (2021). Enhancing waste degradation and biogas production by pre-digestion with a hyperthermophilic anaerobic bacterium. Biofuel Res. J..

[21] Straub C.T., Khatibi P.A., Otten J.K., Adams M.W.W., Kelly R.M. (2019). Lignocellulose solubilization and conversion by extremely thermophilic *Caldicellulosiruptor bescii* improves by maintaining metabolic activity. Biotechnol. Bioeng..

[22] Brunecky R., Donohoe B.S., Yarbrough J.M., Mittal A., Scott B.R., Ding H., Taylor L.E., Russell J.F., Chung D., Westpheling J. (2017). The Multi Domain *Caldicellulosiruptor bescii* CelA Cellulase Excels at the Hydrolysis of Crystalline Cellulose. Sci. Rep..

[23] Bing R.G., Willard D.J., Crosby J.R., Adams M.W.W., Kelly R.M. (2023). Whither the genus *Caldicellulosiruptor* and the order Thermoanaerobacterales: Phylogeny, taxonomy, ecology, and phenotype. Front. Microbiol..

[24] Gu J., Qiu Q., Yu Y., Sun X., Tian K., Chang M., Wang Y., Zhang F., Huo H. (2024). Bacterial transformation of lignin: Key enzymes and high-value products. Biotechnol. Biofuels Bioprod..

[25] Dustin J.S., Hansen C.L. (2012). Completely stirred tank reactor behavior in an unmixed anaerobic digester: The induced bed reactor. Water Environ. Res..

[26] Lee E., Min K.J., Park K.Y. (2025). Changes in anaerobic digestion performance and microbial community by increasing SRT through sludge recycling in food waste leachate treatment. Sci. Rep..

[27] Bonk F., Popp D., Weinrich S., Sträuber H., Becker D., Kleinsteuber S., Harms H., Centler F. (2019). Determination of Microbial Maintenance in Acetogenesis and Methanogenesis by Experimental and Modeling Techniques. Front. Microbiol..

[28] Bolyen E., Rideout J.R., Dillon M.R., Bokulich N.A., Abnet C.C., Al-Ghalith G.A., Alexander H., Alm E.J., Arumugam M., Asnicar F. (2019). Reproducible, interactive, scalable and extensible microbiome data science using QIIME 2. Nat. Biotechnol..

[29] Callahan B.J., McMurdie P.J., Rosen M.J., Han A.W., Johnson A.J.A., Holmes S.P. (2016). DADA2: High-resolution sample inference from Illumina amplicon data. Nat. Methods.

[30] Price M.N., Dehal P.S., Arkin A.P. (2010). FastTree 2—Approximately Maximum-Likelihood Trees for Large Alignments. PLoS ONE.

[31] Quast C., Pruesse E., Yilmaz P., Gerken J., Schweer T., Yarza P., Peplies J., Glöckner F.O. (2013). The SILVA ribosomal RNA gene database project: Improved data processing and web-based tools. Nucleic Acids Res..

[32] Hugerth L.W., Andersson A.F. (2017). Analysing Microbial Community Composition through Amplicon Sequencing: From Sampling to Hypothesis Testing. Front. Microbiol..

[33] Blazewicz S.J., Barnard R.L., Daly R.A., Firestone M.K. (2013). Evaluating rRNA as an indicator of microbial activity in environmental communities: Limitations and uses. ISME J..

[34] Wickham H., Averick M., Bryan J., Chang W., McGowan L.D., François R., Grolemund G., Hayes A., Henry L., Hester J. (2019). Welcome to the tidyverse. J. Open Source Softw..

[35] Oksanen J., Simpson G.L., Blanchet F.G., Kindt R., Legendre P., Minchin P.R., O’Hara R.B., Solymos P., Stevens M.H.H., Szoecs E. vegan: Community Ecology Package 2025. https://cran.r-project.org/web/packages/vegan/index.html.

[36] Griffith D.M., Veech J.A., Marsh C.J. (2016). cooccur: Probabilistic Species Co-Occurrence Analysis in R. J. Stat. Soft..

[37] Fruchterman T.M.J., Reingold E.M. (1991). Graph drawing by force-directed placement. Softw. Pract. Exp..

[38] Pedersen T.L. (2024). ggraph: An Implementation of Grammar of Graphics for Graphs and Networks.

[39] Pyzik A., Ciezkowska M., Krawczyk P.S., Sobczak A., Drewniak L., Dziembowski A., Lipinski L. (2018). Comparative analysis of deep sequenced methanogenic communities: Identification of microorganisms responsible for methane production. Microb. Cell Factories.

[40] Conklin A., Stensel H.D., Ferguson J. (2006). Growth kinetics and competition between *Methanosarcina* and *Methanosaeta* in mesophilic anaerobic digestion. Water Environ. Res..

[41] St-Pierre B., Wright A.-D.G. (2013). Metagenomic analysis of methanogen populations in three full-scale mesophilic anaerobic manure digesters operated on dairy farms in Vermont, USA. Bioresour. Technol..

[42] Yadav S., Koenen M., Bale N.J., Reitsma W., Engelmann J.C., Stefanova K., Damsté J.S.S., Villanueva L. (2024). Organic matter degradation in the deep, sulfidic waters of the Black Sea: Insights into the ecophysiology of novel anaerobic bacteria. Microbiome.

[43] Huber R., Hannig M., Dworkin M., Falkow S., Rosenberg E., Schleifer K.-H., Stackebrandt E. (2006). Thermotogales. The Prokaryotes: Volume 7: Proteobacteria: Delta, Epsilon Subclass.

[44] Johnson M.R., Conners S.B., Montero C.I., Chou C.J., Shockley K.R., Kelly R.M. (2006). The Thermotoga maritima Phenotype Is Impacted by Syntrophic Interaction with *Methanococcus jannaschii* in Hyperthermophilic Coculture. Appl. Environ. Microbiol..

[45] Zinder S.H., Anguish T., Cardwell S.C. (1984). Effects of Temperature on Methanogenesis in a Thermophilic (58 °C) Anaerobic Digestor. Appl. Environ. Microbiol..

[46] Mladenovska Z., Ahring B.K. (2000). Growth kinetics of thermophilic *Methanosarcina* spp. isolated from full-scale biogas plants treating animal manures. FEMS Microbiol. Ecol..

[47] Ni J., Hatori S., Wang Y., Li Y.-Y., Kubota K. (2020). Uncovering Viable Microbiome in Anaerobic Sludge Digesters by Propidium Monoazide (PMA)-PCR. Microb. Ecol..

[48] Kläring K., Just S., Lagkouvardos I., Hanske L., Haller D., Blaut M., Wenning M., Clavel T. (2015). *Murimonas intestini* gen. nov., sp. nov., an acetate-producing bacterium of the family *Lachnospiraceae* isolated from the mouse gut. Int. J. Syst. Evol. Microbiol..

[49] McInerney M.J., Bryant M.P., Hespell R.B., Costerton J.W. (1981). *Syntrophomonas wolfei* gen. nov. sp. nov., an Anaerobic, Syntrophic, Fatty Acid-Oxidizing Bacterium. Appl. Environ. Microbiol..

[50] Yokoyama H., Wagner I.D., Wiegel J. (2010). *Caldicoprobacter oshimai* gen. nov., sp. nov., an anaerobic, xylanolytic, extremely thermophilic bacterium isolated from sheep faeces, and proposal of *Caldicoprobacteraceae* fam. nov. Int. J. Syst. Evol. Microbiol..

[51] Shaw A.J., Hogsett D.A., Lynd L.R. (2009). Identification of the [FeFe]-Hydrogenase Responsible for Hydrogen Generation in *Thermoanaerobacterium saccharolyticum* and Demonstration of Increased Ethanol Yield via Hydrogenase Knockout. J. Bacteriol..

[52] Zavarzina D.G., Tourova T.P., Kuznetsov B.B., Bonch-Osmolovskaya E.A., Slobodkin A.I. (2002). *Thermovenabulum ferriorganovorum* gen. nov., sp. nov., a novel thermophilic, anaerobic, endospore-forming bacterium. Int. J. Syst. Evol. Microbiol..

[53] Hu Y., Shen C. (2024). Thermophilic-mesophilic temperature phase anaerobic co-digestion compared with single phase co-digestion of sewage sludge and food waste. Sci. Rep..

[54] Gallert C., Winter J. (1997). Mesophilic and thermophilic anaerobic digestion of source-sorted organic wastes: Effect of ammonia on glucose degradation and methane production. Appl. Microbiol. Biotechnol..

[55] Sasaki K., Morita M., Sasaki D., Nagaoka J., Matsumoto N., Ohmura N., Shinozaki H. (2011). Syntrophic degradation of proteinaceous materials by the thermophilic strains *Coprothermobacter proteolyticus* and *Methanothermobacter thermautotrophicus*. J. Biosci. Bioeng..

[56] Hagen L.H., Frank J.A., Zamanzadeh M., Eijsink V.G.H., Pope P.B., Horn S.J., Arntzen M.Ø. (2016). Quantitative Metaproteomics Highlight the Metabolic Contributions of Uncultured Phylotypes in a Thermophilic Anaerobic Digester. Appl. Environ. Microbiol..

[57] Dong N., Bu F., Zhou Q., Khanal S.K., Xie L. (2018). Performance and microbial community of hydrogenotrophic methanogenesis under thermophilic and extreme-thermophilic conditions. Bioresour. Technol..

[58] Maus I., Cibis K.G., Bremges A., Stolze Y., Wibberg D., Tomazetto G., Blom J., Sczyrba A., König H., Pühler A. (2016). Genomic characterization of *Defluviitoga tunisiensis* L3, a key hydrolytic bacterium in a thermophilic biogas plant and its abundance as determined by metagenome fragment recruitment. J. Biotechnol..

[59] Miller T.L., Wolin M.J. (1985). *Methanosphaera stadtmaniae* gen. nov., sp. nov.: A species that forms methane by reducing methanol with hydrogen. Arch. Microbiol..

